# First steps in establishing surveillance of bloodstream infections from electronic health record derived data, EU/EEA countries, March 2023 to March 2025

**DOI:** 10.2807/1560-7917.ES.2026.31.25.2500618

**Published:** 2026-06-25

**Authors:** Alexis Sentís, Tine Dalby, Tommi Κärki, Francisco Orchard, Angelo D'Ambrosio, Miriam Van den Nest, Bernhard Benka, Nathalie Shodu, Karl Mertens, Emanuel Brađašević, Antea Jezidžić, Markella Marcou, Linos Hadjihannas, Jan Kubele, Sophie Gubbels, Louise Stougaard, Liidia Dotsenko, Piret Mitt, Imke Wieters, Alexandra Hoffmann, Antonis Maragkos, Fortunato D'Ancona, Agnese Comelli, Auguste Salnaite, Andre Brincat, Michael A Borg, Manon Brekelmans, Stephanie van Rooden, Anders Skyrud Danielsen, Torunn Alberg, Irena Klavs, Daša Kavka, Lucía García-San Miguel, Pilar Gallego-Berciano, Hanna Billström, Olov Aspevall, Diamantis Plachouras, Hanne-Dorthe Emborg, Carlos Carvalho, Anthony Nardone, on behalf of the EHR-BSI working group

**Affiliations:** 1 Epiconcept, Paris, France; 2 Statens Serum Institut (SSI), Infectious Disease Epidemiology & Prevention, Copenhagen, Denmark; 3 European Centre for Disease Prevention and Control (ECDC), Stockholm, Sweden; 4 Austrian Agency for Health and Food Safety, Institute for Surveillance and Infectious Disease Epidemiology, Vienna, Austria; 5 Austrian Agency for Health and Food Safety, Division Public Health, Vienna, Austria; 6 Sciensano, Brussels, Belgium; 7 Croatian Institute of Public Health, Division for Medical Informatics and Biostatistics, Zagreb, Croatia; 8 ECDC Fellowship Programme, Field Epidemiology Path (EPIET), European Centre for Disease Prevention and Control (ECDC), Stockholm, Sweden; 9 Medical and Public Health Services, Cyprus Ministry of Health, Nicosia, Cyprus; 10 National Institute of Public Health (NIPH), Prague, Czechia; 11 Statens Serum Institut (SSI), Department of Data Integration and Analysis, Copenhagen, Denmark; 12 Department of Communicable Diseases Epidemiology, Health Board, Tallinn, Estonia; 13 Tartu University Hospital, Tartu, Estonia; 14 Robert Koch Institute, Berlin, Germany; 15 Hellenic Centre for Disease Control and Prevention, Athens, Greece; 16 Istituto Superiore di Sanità, Rome, Italy; 17 Infectious Disease Consultant, General Directorate for Health, Lombardy Region, Milan, Italy; 18 Institute of Hygiene, Vilnius, Lithuania; 19 Mater Dei Hospital, Msida, Malta; 20 Centre for Infectious Diseases Control, National Institute for Public Health and the Environment, Bilthoven, the Netherlands; 21 Department of Medical Microbiology and Infection Control, University Medical Centre Utrecht, Utrecht, the Netherlands; 22 Department of Infection Control and Preparedness, Norwegian Institute of Public Health (NIPH), Oslo, Norway; 23 National Institute of Public Health (NIJZ), Ljubljana, Slovenia; 24 National Laboratory for Health, Environment and Food (NLZOH), Ljubljana, Slovenia; 25 Coordination Centre for Health Alerts and Emergencies, Ministry of Health, Madrid, Spain; 26 Public Health Agency of Sweden, Solna, Sweden; 27 The members of the EHR-BSI working group are listed under Acknowledgements

**Keywords:** Healthcare-associated infections, Bloodstream infection, Electronic Health Records, Public health surveillance

## Abstract

**BACKGROUND:**

Enhancing surveillance of bloodstream infections (BSIs) by extracting data from electronic health records (EHR) is often the first step in automating the surveillance of healthcare-associated infections (HAIs).

**AIM:**

We assessed existing BSI surveillance systems and the progress in implementing EHR-BSI surveillance within the ECDC project Surveillance from Electronic Health Data (SUREHD).

**METHODS:**

We summarised information on BSI surveillance systems through meetings with national representatives and review of national surveillance protocols and reports.

**RESULTS:**

Of the 30 EU/EEA countries invited to join the SUREHD project, 17 actively participated from March 2023 to March 2025. All 17 countries conducted BSI surveillance within HAI or antimicrobial resistance programmes, and EHR-BSI surveillance was being implemented nationally (n = 9), regionally (n = 2) or at hospital level (n = 6). Reported challenges included data standardisation (n = 13), IT capacity (n = 10), data linkage (n = 7), system integration (n = 7) and data protection (n = 6). Few countries had access to structured EHR data on patient symptoms (n = 1), infection origin (n = 2) and catheter-related variables (n = 5). Most countries had not yet implemented data validation processes (n = 12) nor decided on standardised vocabularies for most variables. All countries aimed to automate EHR-BSI surveillance.

**CONCLUSION:**

Automation of BSI surveillance is critically relevant for the development of surveillance systems and to support infection and control measures (IPC) for HAIs. Implementation of automated EHR-based BSI surveillance varied between countries; a generic protocol and a network of experts have established a way forward. Future efforts should focus on harmonisation, data quality, and leveraging EHR-BSI data to strengthen HAI surveillance and to improve IPC.

**Key public health message**

**What did you want to address in this study and why?**

As opposed to traditional reporting, automated data extraction and processing from electronic health records (EHR) can provide more timely and easily accessible data on diseases or infections to inform prevention and control strategies. Here, we describe a European project aimed at using EHR for surveillance of bloodstream infections (BSI) and moving towards comparable data across 17 countries in the EU/EEA.

**What have we learnt from this study?**

All participating countries reported similar challenges in the implementation of automated or semi-automated EHR-based BSI surveillance systems, including data standardisation, insufficient IT capacity and data protection issues. There is a need in most countries to advance and implement standardised controlled vocabularies, common data models and validation processes.

**What are the implications of your findings for public health?**

The implementation of EHR-based surveillance systems for BSI will be essential for improving data harmonisation and quality. This project has supported the implementation and strengthening of automated EHR-based BSI surveillance across Europe. The surveillance data can ultimately lead to better infection prevention and control measures for BSI at local, national and EU levels.

## Introduction

The incidence of bloodstream infections (BSIs) is one of the measures traditionally used to monitor targets for the prevention and control of healthcare-associated infections (HAIs) [1]. Bloodstream infections are of increasing public health concern, mainly due to an ageing population, the use of immunosuppressive therapies and antimicrobial resistance (AMR), which complicate treatment [2-5]. In western countries, BSI incidence has risen over the past decades, with annual rates ranging from 122 to 300 cases per 100,000 people [2,5]. Estimates indicate that nosocomial BSIs account for ca 30–50% of BSI cases in high-income countries [6]. Data from Finland have shown monthly case fatality rates for nosocomial BSIs ranging from 17% to 28% and for community-acquired BSIs from 10% to 19% [2].

The use of electronic health record (EHR) data for surveillance of BSI through automatic extraction and case identification can improve traditional BSI surveillance built on review of patient records and subsequent notification [7,8]. Nevertheless, EHR-based surveillance systems require robust protocols that describe the data sources and methods for data linkage to harmonise case definitions employed in the different data sources. Well-structured databases, along with common data models, are essential to ensure interoperability. Additionally, semi-automated and fully automated systems for data extraction should be implemented, supported by strong data security measures and appropriate ethical approvals. Surveillance systems using EHR have been implemented in several European countries, and growing evidence supports their benefits for public health surveillance, including improvements in flexibility, timeliness, data completeness, sustainability and coverage compared with traditional methods [7-10].

The European Centre for Disease Prevention and Control (ECDC) project Surveillance from Electronic Health Data (SUREHD) from 2022 to 2026 aimed to support European Union/European Economic Area (EU/EEA) countries in piloting, implementing and evaluating surveillance systems using EHR data for three infectious diseases or conditions [11]: severe acute respiratory infections (SARI, launched in October 2022), bloodstream infections (BSI, launched in March 2023) and sexually transmitted infections (STI, launched in March 2024) [11,12].

We describe the BSI surveillance systems of 17 EU/EEA countries actively participating in the first 2 years of the project from March 2023 to March 2025, along with the progress and challenges in implementing EHR-based BSI surveillance in that period.

## Methods

In December 2022, ECDC asked through the ECDC Coordinating Competent Body structure (National Coordinators and National Focal Points for HAIs and Surveillance) for expressions of interest from the 30 EU/EEA countries in participating in the SUREHD EHR-BSI project [13]. Although the number of active countries fluctuated during the implementation of the project, from March 2023 to March 2025, 17 countries were participating in the EHR-BSI project and were included in this study. Nine countries were observers.

### Sources of information

During the first 2 years of the project (study period from March 2023 to March 2025), the study coordination team collected information on existing and planned BSI surveillance systems through a review of surveillance protocols and reports from 17 countries prepared as part of the SUREHD EHR-BSI project and through regular meetings with the participating countries.

### Standardised template to build country profiles

In this study, we defined EHR-based surveillance activities as the use of electronic health data related to a natural person, collected and processed within the healthcare system for the purpose of the provision of healthcare [14].

We systematically organised and summarised collected information from the 17 countries into country profiles using a standardised template of 32 components. The full subject wording, the categories for sublevels as well as all 17 pseudonymised profiles from countries are presented in Supplementary Table S1. The resulting country profiles were approved by each country and comprised components divided into two main sections: the existing BSI surveillance system, and the plans and anticipated advances in their EHR-BSI surveillance systems under development. The description of the existing BSI surveillance systems at the start of the study period, as assessed by the study coordination team, included information on surveillance objectives, whether surveillance was mandatory or voluntary, the microorganisms under surveillance, and the levels of data aggregation (e.g. by hospital, region or unit speciality) for reporting to either national or ECDC-level surveillance systems.

In the country profile template concerning EHR-based BSI surveillance systems under development, we included planned surveillance objectives, key system characteristics, available data sources and variables, case and episode definitions, data processing and standardisation, patient linkage across EHR systems, denominator data, legal issues and plans for data sharing with ECDC. A description of validation processes was also included, referring to both the countries' plans for ensuring the quality of the collected data and for confirming the validity of BSI cases.

The challenges faced during the implementation were addressed, as well as the readiness of the national EHR systems for EHR-based BSI surveillance by March 2025. The prerequisites for the implementation of a surveillance data flow were studied, starting with the availability of the required data in hospitals and laboratories, the strategies and methods used to identify cases, and the existence of national protocols and technologies for data collection and standardisation. Countries were asked about their IT solutions adopted for data collection, specifically whether a dedicated tool was installed at each site or a central application programming interface (API) was provided. Information on the adoption of international standards was also collected, including use of coding systems (e.g. Systematised Nomenclature of Medicine-Clinical Terms (SNOMED-CT), Logical Observation Identifiers Names and Codes (LOINC), International Statistical Classification of Diseases and Related Health Problems (ICD)), analytical models (e.g. the Observational Medical Outcomes Partnership Common Data Model (OMOP CDM)) and health data exchange standards (e.g. Health Level Seven (HL7), Fast Healthcare Interoperability Resources (FHIR)).

### Definitions for bloodstream infections and healthcare-associated bloodstream infections

As part of the SUREHD activities, we prepared a generic protocol for EHR-based surveillance of BSI to assist the participating countries in developing standardised systems. The countries were not obliged to follow the exact definitions but were encouraged to adopt them. Later, ECDC developed a reporting protocol for reporting of data through the European Surveillance System (TESSy) using the same data model and variables as in the generic protocol. An abridged version of the generic protocol is presented in Supplementary material 2. The two protocols were available to all participating countries, and the corresponding data model is now publicly accessible in the TESSy metadata set [15]. The BSI case definition was adapted from the EU case definition [16]. The protocols proposed definitions for a BSI case, an episode of BSI and healthcare (HA-BSI) vs community-associated (CA-BSI) BSI episodes (Box).

Box **Definitions of bloodstream infections and episodes of bloodstream infections**

**Bloodstream infection (BSI):**

• Detection of a recognised pathogen from blood culture

OR

• Detection of the same species or subtype of a common skin contaminant from two separate blood cultures obtained within 3 days^a^.

**Healthcare-associated BSI (HA-BSI):**

• BSI associated with a healthcare visit.

• Hospital-onset (HO-BSI):

o Onset of symptoms or isolation of a pathogen from blood culture ≥ 3 days after a hospital admission^a^.

• Imported HA-BSI:

o Date of symptom onset or isolation of a pathogen from blood culture ≤ 3 days after discharge from a healthcare facility^a^.

**Community-associated BSI (CA-BSI):**

• Onset of symptoms or isolation of a pathogen from blood culture < 3 days after admission to a healthcare facility or > 3 days after discharge from a healthcare facility^a^.

**Episode of BSI:**

• A 14-day period from the date of onset or from the date of the first positive blood culture.

• Rolling 14-day window if the same species or subtype is identified in a blood culture within an episode.

• Polymicrobial episode

o > 1 species/subtype isolated from a blood culture < 3 days from the date of onset or from the date of the first positive blood culture^a^.

o In case of a different species/subtype being isolated from a blood culture ≥3 days after onset, this will count as a new episode.

^a^ In the date comparisons for within 3 days and < or > 3 days, date of specimen/admission/discharge = day one.

As reporting practices may vary across countries, particularly in terms of the definition of episodes, information was collected about the episode duration used within each country.

Denominators were defined as either the number of discharges (or admissions), the number of patient days or the size of the catchment population.

## Results

Altogether 17 EU/EEA countries participated in the study: Austria, Belgium, Croatia, Cyprus, Czechia, Denmark, Estonia, Germany, Greece, Italy, Lithuania, Malta, the Netherlands, Norway, Slovenia, Spain and Sweden. Pseudonymised 32-component profiles by country can be found in Supplementary Table S1. Nine additional EU/EEA countries were observers.

During the study period, all 17 participating countries implemented or improved EHR-based BSI surveillance activities at the hospital, regional or national level with the aim of progressing towards a more standardised, interoperable and automated national BSI surveillance system using EHR data.

### Main characteristics of the existing surveillance systems for bloodstream infections

All 17 countries included in this study reported BSI surveillance activities as part of HAI and/or AMR monitoring programmes when the SUREHD project for EHR-BSI started in March 2023. Even though these activities often relied on electronic health data, none could be considered as automated national EHR-based BSI surveillance including pathogen information. As stated by the countries, the main objective of BSI-related data collection was to monitor BSI at the country level while contributing to EU surveillance initiatives, such as the European Antimicrobial Resistance Surveillance Network (EARS-Net) [17] (n = 10) and Healthcare-associated Infections Surveillance Network (HAI-Net) [18] (n = 7) (Table 1). Additionally, nine countries cited AMR surveillance as an objective for collecting BSI data. In nine countries, BSI surveillance was voluntary, while mandatory in eight countries. Nine countries reported surveillance data only for a selected country-specific list of pathogens. Data aggregation commonly included reporting by geography (n = 13) and/or by hospital/hospital type (n = 10).

**Table 1:** Main characteristics of the existing surveillance systems for bloodstream infections, European Union/European Economic Area (EU/EEA) countries, March 2023–March 2025 (n = 17)

Characteristic^a^	n
Main objectives (multiple selection)	
Country monitoring while participating in EU/EEA EARS-Net	10
National/regional/local AMR surveillance	9
Country monitoring of BSIs while participating in EU/EEA HAI-Net (e.g. PPS)	7
National/regional/local BSI surveillance	4
National/regional/local HAI surveillance	3
Mandatory/voluntary BSI surveillance (single selection)	
Voluntary for BSI attributed to a specific list of pathogens	5
Voluntary for BSI attributed to any pathogen	4
Mandatory for BSI attributed to a specific list of pathogens	4
Mandatory for BSI attributed to any pathogen	4
Aggregation of published surveillance data (multiple selection)	
Geography (e.g. hospital catchment area, municipality or region)	13
Hospital or type of hospital (e.g. tertiary hospital)	10
Sex	8
Age group	8
Ward/speciality	5
HO-BSI or HA-BSI vs non-HO-BSI/non-HA-BSI	3
Other	2

AMR: antimicrobial resistance; BSI: bloodstream infection; EARS-Net: European Antimicrobial Resistance Surveillance Network; HAI: healthcare-associated infection; HA-BSI: healthcare-associated BSI; HAI-Net: Healthcare-associated Infections Surveillance Network; HO-BSI: hospital-onset BSI; PPS: point prevalence survey.

^a^ In multiple selection, countries could select several applicable options. In single selection, only one option could be chosen per country.

### Main characteristics and challenges of surveillance systems for bloodstream infections using electronic health record data

The EHR-BSI surveillance was planned to be national in nine countries, run in 1–16 selected hospitals in six countries and regional in two countries (two and three regions, respectively) (Table 2). Common EHR data sources were laboratory information management systems (LIMS) (n = 15) and hospital databases (n = 14). Of these countries, 12 were planning to use both data from LIMS and hospital databases. The primary denominators for calculating BSI incidence were patient days (n = 14), followed by catchment population (n = 8) and number of admissions/discharges (n = 7), with four countries using all three denominators. Furthermore, 10 countries were able to calculate BSI incidence by ward or speciality, while two countries calculated by intensive care units (ICU) and non-ICU.

**Table 2:** Main characteristics and challenges of surveillance systems under development for bloodstream infections using electronic health record data, European Union/European Economic Area (EU/EEA) countries, March 2023–March 2025 (n = 17)

Characteristic^a^	n
Coverage of EHR-BSI surveillance (single selection)	
National	9
Selected hospitals	6
Selected regions	2
Data sources that will be used for EHR-BSI surveillance (multiple selection)	
Laboratory information management systems	15
Hospital databases	14
Antimicrobial resistance surveillance	3
Administrative databases	3
Insurance databases	1
Other^b^	4
Incidence denominators (multiple selection)	
Patient days	14
Catchment population	8
Discharges/admissions	7
All of the above	4
Denominators aggregated by ward (single selection)	
Yes	10
Yes (only in two categories: ICU/non-ICU)	2
No	4
Unknown	1
Episode duration (single selection)	
14 days (episode time frame from day 0 to day 13)	12
30 days (episode time frame from day 0 to day 29)^c^	4
Not possible to identify episodes	1
HA-BSI incidence monitoring frequency (single selection)	
Yearly	2
Bi-annually	1
Quarterly	9
Weekly	1
Near real-time	4
Pathogens planned to be under surveillance (single selection)	
All pathogens (bacteria and fungi)	11
Only EARS-Net microbes	3
Selected national list	2
All bacteria	1
Possible to identify HO-BSI and imported HA-BSI (single selection)	
Yes, both	9
Yes, HO-BSI	7
No	1
Main challenges (multiple selection)	
Health data standardisation	13
Other (limited IT resources/capacity)	10
Integration of systems	7
Data linkage (technical)	7
Digitalisation	6
Data protection compliance issues (including data linkage due to GDPR)	6

BSI: bloodstream infection; EARS-Net: European Antimicrobial Resistance Surveillance Network; HER: electronic health record; GDPR: General Data Protection Regulation; HA-BSI: healthcare-associated BSI; HO-BSI: hospital-onset BSI; ICU: intensive care unit.

^a^ In multiple selection, countries could select several applicable options. In single selection, only one option could be chosen per country.

^b^ Information provided via email, from national patient registries, software tools developed for antimicrobial consumption and antimicrobial stewardship programmes.

^c^ Countries were not obliged to follow the exact project definitions but were encouraged to adopt them.

Twelve countries used 14 days (days 0–13), while four countries used 30 days (days 0–29) as the maximum number of days between positive blood cultures with the same pathogen to count as the same episode (Table 2). Eleven countries reported BSIs from all pathogens, three reported only BSIs caused by the eight bacterial pathogens included in EARS-Net (*Escherichia coli, Klebsiella pneumoniae, Pseudomonas aeruginosa, Acinetobacter* species, *Streptococcus pneumoniae, Staphylococcus aureus, Enterococcus faecalis, Enterococcus faecium*), two according to a national list of pathogens, and one country reported only bacterial BSIs. Nine countries collected admission and discharge dates to identify imported HA-BSI, whereas seven only collected admission dates and identified hospital-onset BSI (HO-BSI). For HA-BSI incidence monitoring, quarterly data collection and analysis was the most common planned periodicity (n = 9).

The main challenges countries faced in implementing EHR-BSI monitoring were standardisation (n = 13), limited IT capacity (n = 10), systems integration (n = 7) and data linkage (n = 7). Data protection compliance (n = 6) and digitalisation (n = 6) were additional key issues (Table 2).

### Availability of data stored in a structured way in surveillance systems for bloodstream infections using electronic health record data

Countries reported varying degrees of availability of structured EHR data by different groups of variables, ranging from maximum availability for basic patient characteristics (all countries) and patient admissions and discharges (n = 16) to minimum availability for the origin of infection (n = 2), patient signs and symptoms (n = 1) and catheter insertion and removal (n = 5) (Table 3). Nevertheless, several countries had some data stored as unstructured free text in their EHR, mostly for patient signs and symptoms (n = 9) and for catheter procedures (n = 5).

**Table 3:** Availability of health data stored in a structured way in electronic health record data systems, by variable group, European Union/European Economic Area (EU/EEA) countries, March 2023–March 2025 (n = 17)

Variable group	Structured (n)	Free text (n)	Not available (n)
Patient characteristics	17	0	0
Patient admissions and discharges	16		1
Hospital unit (ward/speciality)	16		1
Hospital type (primary, secondary, tertiary)	16		1
Blood culture performed and results	14		3
Susceptibility tests performed and AST results	14		3
Patient main diagnosis codes	14		3
Denominators	14		3
Patient origin/transfer	12		5
Outcome (death)	11	1	5
Catheter procedures	5	5	7
Origin of infection	2	1	14
Patient signs and symptoms	1	9	7

AST: antimicrobial susceptibility testing.

### Data processing, linkage, exchange and level of automation of the surveillance systems under development for bloodstream infections using electronic health record data

In 11 countries, data linkage between sources was possible for EHR-BSI surveillance using patient identifiers (Table 4). One country could partially link their data. However, in five countries, legal issues (including General Data Protection Regulation (GDPR)) or technical limitations prevented such linkage. Data linkage occurred, or was expected to occur, either nationally (n = 9), locally (n = 5) or regionally (n = 2) or in a combination. Three countries did not expect that linkage would soon be possible. In countries where this had already been decided, the most common IT tool for mapping and extracting EHR data was an ad hoc central API, allowing sites to submit data (n = 7). Some countries reported using or considering the FHIR standard (n = 5) for data collection. However, seven countries had not yet finalised the specification of their data collection process.

**Table 4:** Data processing, linkage, exchange and level of automation of the surveillance systems under development for bloodstream infections using electronic health record data, European Union/European Economic Area (EU/EEA) countries, March 2023–March 2025 (n = 17)

Characteristic^a^	n
Data linkage possible (single selection)	
Yes	11
Yes, partially	1
No, due to technical limitations/issues	2
No, due to both legal and technical limitations	2
No, by legal limitations (or related to GDPR issues)	1
Data linkage level in the foreseen system (multiple selection; highest level shown)	
National	9
Local level	5
Regional level	2
No data linkage	3
Data collection approach (multiple selection)	
Data collection specification to be finalised	7
A nationally developed central API will be used to allow sites to submit data in a given format	7
An international standard FHIR (or equivalent) will be implemented at sites to extract data from hospitals or laboratories	5
A given data extraction software will be installed by hospitals or laboratories to collect data	1
A secured file server will be used to submit data to a central level	1
HL7 FHIR profiles (or equivalent) will be used for EHR sources	0
Expected level of automation (multiple selection)	
Data extraction/data collection	17
Data linkage/merging datasets	11
BSI case/episode identification	11
Data exchange/reporting	8
Identifying two cultures with skin contaminants (48 h or three calendar days in between)	6
Denominator calculation	6
Triggering of alerts within the surveillance/monitoring system	2
Validation processes (single selection)	
Validation processes planned to be defined during the timespan of the project (March 2023 to March 2026)	12
Validation processes well-defined but need to be manually performed	4
Data quality is already validated so no manual validation is needed	1
Validation processes are not well-defined	0
Possible granularity of data shared (single selection)	
Both case-based and aggregated	5
Case-based	3
Aggregated	7
Not decided yet	2

API: application programming interface; BSI: bloodstream infection; EHR: electronic health record; FHIR: Fast Healthcare Interoperability Resources; GDPR: General Data Protection Regulation; HL7: Health Level Seven.

^a^ In multiple selection, countries could select several applicable options. In single selection, where only one option could be chosen per country.

In developing and improving EHR-BSI surveillance, countries aimed to attain a high level of automation. Many were transitioning from manual or semi-automated systems with plans to increase automation across various components. All countries expected to automate data extraction, 11 anticipated automating data linkage and eight planned for automated data exchange/reporting (Table 4). Eleven countries planned to automate the identification of cases/episodes, while the remaining six expected to still rely on manual identification. Few countries planned to automate the detection of BSI caused by common skin contaminant bacteria (n = 6), denominator calculation (n = 6) or triggering of alerts (n = 2). Most countries planned to define validation processes during the project (n = 12) or have manual processes in place (e.g. validation of data through expert review) (n = 4).

All 17 countries planned to share their EHR-BSI surveillance data with ECDC. Five expected to share both case-based and aggregated data, three would share case-based data, seven only aggregated data, and two were undecided (Table 4).

### Data standardisation: data model and standard vocabularies/terminologies planned to be used in surveillance systems for bloodstream infections using electronic health record data

Data available from the EHR systems were mostly stored using a nationally developed schema (n = 15), while two countries reported using an OMOP common data model [19]. For most variable groups, the vocabularies/terminologies planned to be used to standardise EHR-based data were unknown in several countries (n = 8 for patient variables and n = 12 for catheter procedures), either because vocabularies had not yet been decided or details were not provided (Table 5). For microorganisms, the vocabularies that countries most frequently used or planned to use were SNOMED-CT (n = 8), WHOCARE [20] (n = 4) and LOINC (n = 4). For antimicrobials, EARS-Net coding [17] was the category most frequently used by countries (n = 4), followed by the Anatomical Therapeutic Chemical (ATC) classification and LOINC (n = 3 in both cases) (Table 6).

**Table 5:** Standard vocabularies or terminologies planned to be used for patient variables, diagnostics, and catheter procedures in surveillance systems under development for bloodstream infections using electronic health record data, European Union/European Economic Area (EU/EEA) countries, March 2023–March 2025 (n = 17)

Vocabulary	Patient variables^a^	Diagnostics	Catheter procedures
International Classification of Diseases (ICD)	NA	12	NA
ICD-9	NA	1	NA
ICD-10	NA	8	NA
ICD-11	NA	3	NA
SNOMED-CT	3	3	2
National vocabulary	1	NA	1
Unknown (no details provided)	8	3	12
Other	5	1	2

NA: not applicable; SNOMED-CT: Systematised Nomenclature of Medicine – Clinical Terms.

^a^ Patient variables: clinical (not including diagnostics), administrative (admission, discharge) and sociodemographic (sex, age) variables.

**Table 6:** Standard vocabularies or terminologies planned to be used for microorganisms and antimicrobials in the surveillance systems for bloodstream infections using electronic health record data, European Union/European Economic Area (EU/EEA) countries, March 2023–March 2025 (n = 17)

Vocabulary^a^	Variable groupings	
	Microorganisms	Antimicrobials
SNOMED-CT	8	NA
LOINC	4	3
WHOCARE	4	2
National vocabulary	1	NA
NCBI	1	NA
NPU (locally adapted)	1	1
EARS-Net	NA	4
ATC	NA	3
Unknown (no details provided)	1	4
Other	1	2

ATC: Anatomical Therapeutic Chemical Classification; EARS-Net: European Antimicrobial Resistance Surveillance Network; LOINC: Logical Observation Identifiers Names and Codes; NA: not applicable; NCBI: National Center for Biotechnology Information; NPU: Nomenclature for Properties and Units Laboratory Terminology; SNOMED-CT: Systematised Nomenclature of Medicine – Clinical Terms; WHOCARE: WHOCARE organism codes are a semi-standardised microbiology coding convention historically used TESSy and in WHONET-compatible antimicrobial resistance surveillance datasets [20].

## Discussion

We assessed the existing BSI surveillance systems and the development and implementation of EHR-BSI surveillance systems in 17 European countries. At the start of the SUREHD project in March 2023, while all participating countries were monitoring BSI within pre-existing HAI and AMR surveillance programmes, none of them had an established automated national EHR-based surveillance system for BSIs. Such an automated system can play an essential role in the timely monitoring of BSIs and thereby contributing to reducing morbidity and mortality due to BSIs. The WHO guidelines state that HAI and AMR surveillance, including BSI monitoring, are a core component of infection prevention and control (IPC) programmes [21]. Moreover, surveillance of BSI is particularly important to support preventing central line-associated BSIs [22].

Implementation or improvement strategies for EHR-BSI surveillance systems varied, with some countries commencing with national coverage while others beginning at sub-national levels with plans for future expansion. The data collection frequency was initially set at periodic intervals, such as quarterly, but was often planned to transition to real-time reporting as automation capabilities would improve. Although the goal in most countries is comprehensive pathogen monitoring, many countries initially focused on European (e.g. EARS-Net pathogens) or nationally prioritised lists to facilitate implementation.

Most countries used LIMS and hospital databases as their main EHR data sources. Critical variables, such as infection origin, catheter-related data and clinical symptoms were largely unavailable, while infection outcomes and associated diagnoses were accessible in only half of the countries limiting the comprehensiveness and utility of surveillance data. The fact that most countries could report data at various geographical (national-subnational) and hospital levels, and many even by ward/speciality, is pivotal as it facilitates identifying settings with a higher risk of BSI to design, implement or improve effective IPC interventions [23,24].

It is relevant to note that BSI case and episode definitions were adapted to data availability in each country during the set-up of the EHR surveillance systems. An essential first step involved understanding data collection procedures, coding practices and assessing data quality. Although seven countries had not yet determined their IT approach to mapping and collecting data from EHR systems, most planned to adopt either a central API for data submission using ad hoc technologies or an industry standard for health data exchange, such as FHIR, which can enable direct central querying of targeted data [25]. These approaches to syntactic standardisation at the national level are necessary to ensure machine readability and facilitate the seamless exchange of data [26].

Three main challenges for EHR-BSI surveillance were identified by most participating countries: data standardisation, integration of systems, and limited capacity and resources (mostly related to a lack of available IT experts). In many cases, countries had different data sources (e.g. laboratory and clinical) or surveillance systems (e.g. AMR or HAI), which need to be integrated and often used different nomenclatures at local or regional level. Many considered the development of national platforms or interfaces as a solution, where different data can be linked, collected, stored and extracted as needed for different purposes, including BSI surveillance. Most countries saw a need for IT professionals skilled in standardising and integrating systems. Health data standards enhance data quality, usability and reuse, ensuring proper utilisation and interoperability (information can be exchanged and understood between different systems) while requiring time and expertise for staff training and compliance [27]. To address these and other challenges, the European Commission offers funding that can be used to support epidemiological surveillance through several major programmes [28].

The participating countries were all engaged in standardisation and the development of a data model and were interested in sharing experiences with other countries and agreeing on international standards. Nationally, some countries had initiatives that formed interdisciplinary teams to evaluate and select the most effective data models, aligning them with international standards for national and global implementation [29]. At the European level, since 2019, the European eHealth Network (eHN) [30] has worked on a semantic strategy; one of its objectives is to develop a common European EHR exchange format (EEHRXF) [31] and ‘semantic artefacts’ for five priority areas (domains): patient summary, e-prescriptions/e-dispensations, laboratory (medical tests) results, medical imaging and reports. The suitability of these definitions to support EHR-based surveillance has yet to be established.

Closely related to these challenges are full digitalisation and automation, both of which were also identified as challenges by many countries. Automation is one of the main objectives that countries aim to achieve by moving to an EHR-based surveillance system, with all participating countries progressing towards automating data extraction. Validation processes were planned in 12 countries, while four still had defined manual procedures that remain time-consuming. These processes are challenging to plan or perform since clinical confirmation and high data quality assurance are essential to categorise some BSI cases (e.g. severely ill patients who have multiple positive blood cultures). In a recent collaboration between four European hospitals, an algorithm was developed to identify hospital-onset bacteraemia and fungaemia (HOB) using retrospective cohort data, providing a template for automated HOB monitoring [32].

Data linkage is essential for EHR systems, particularly for their primary use in care provision. Ideally, EHR-based surveillance should leverage this linkage, rather than relying on the subsequent linking of data for secondary purposes such as BSI surveillance. Nevertheless, in many participating countries, linking patient data across laboratories, hospitals and regions was still challenging for technical and/or legal reasons. Most countries had a unique patient ID at the national, regional, hospital or laboratory level, allowing linkage of laboratory and clinical data, but local regulations may prevent the linkage at the national level for the purpose of surveillance.

This analysis based on a standardised 32-component country profile provides critical insights into the potential and the barriers to implementation of EHR-BSI surveillance across EU countries. One of the main limitations of our country profile template is that the use of categorised responses with a limited number of options may have resulted in the collection of insufficient contextual and detailed information. However, we believe that the country profiles have generated a general overview of the strategy and current status of the EHR-BSI implementation. This can serve as a first step to support the emergence of methodological consensus and promote further development of standardised and integrated surveillance systems. Another limitation is the heterogeneity of EHR-BSI surveillance systems being developed by participating countries about key characteristics (e.g. pathogens under surveillance). This heterogeneity has been reported by other investigators [33] and as recommended, we have provided information on data sources, surveillance methods and implementation status to support standardisation and cross-country comparisons. Despite these limitations, we believe that our findings provide information on national strategies and highlight differences and gaps between countries during the implementation of their EHR-based surveillance systems.

The value of national EHR-BSI surveillance systems is demonstrated by the priority in all 17 countries on implementing EHR-BSI surveillance nationally. All countries were moreover aiming to automate the surveillance processes for use in improvement of infection prevention and control. The SUREHD project has been instrumental in supporting the implementation of EHR-based surveillance of BSIs at the national and, consequently, at EU/EEA level. Future developments for EHR-BSI surveillance at the national and European levels should ensure continued funding and prioritise harmonising data models, improve data timeliness and quality, and leverage EHR-derived BSI information to enhance HAI surveillance and IPC strategies.

## Conclusion

Within the SUREHD project, 17 EU/EEA countries have progressed towards automated surveillance of BSI using data from EHRs. A European generic protocol for BSI surveillance was developed which has enabled the reporting of harmonised data at both EU/EEA and national levels. Harmonised definitions and variables across hospitals and regions within a country will assist in the development of comprehensive national surveillance systems and, ultimately, lead to comparable surveillance data throughout EU/EEA countries. Enhanced and timely surveillance data may lead to a better understanding of the epidemiology of HAIs within countries and thereby lead to improved measures for IPC.

## Data Availability

Data are presented in aggregated form in the manuscript. The individual profiles for the 32-component template by each country are available in pseudonymised form in the Supplementary material 1, Table S1. Due to the sensitive nature of the profiles, non-pseudonymised data cannot be shared.

## Supplementary Data

1239000 Supplementary Material
