# Supplementary material for: First steps in establishing surveillance of bloodstream infections from electronic health record derived data, EU/EEA countries, March 2023 to March 2025
Source: Euro Surveill. 2026 Jun 25;31(25):2500618. doi: 10.2807/1560-7917.ES.2026.31.25.2500618 (PMC13309757; doi:10.2807/1560-7917.ES.2026.31.25.2500618)
Supplement: Supplementary Material [file 25-00618_SENTIS_Supplement.pdf]

This supplementary material is hosted by Eurosurveillance as supporting information alongside the article **[First steps in establishing surveillance of bloodstream infections from electronic health record derived data, EU/EEA countries, March 2023 to March 2025]**, on behalf of the authors, who remain responsible for the accuracy and appropriateness of the content. The same standards for ethics, copyright, attributions and permissions as for the article apply. Supplements are not edited by Eurosurveillance and the journal is not responsible for the maintenance of any links or email addresses provided therein.

Supplementary material 1: 12 pages

Table S1. Standardised template - Tool to develop countries' profiles and anonymised results by country.

Supplementary material 2: 13 pages

EHR-BSI surveillance generic protocol (abridged version).

Supplementary material 1. Table S1. Standardised template - Tool to develop countries' profiles and anonymised results by country

Table number in text	Subject/type of question	Category	Count	Country 1	Country 2	Country 3	Country 4	Country 5	Country 6	Country 7	Country 8	Country 9	Country 10	Country 11	Country 12	Country 13	Country 14	Country 15	Country 16	Country 17
**I. Current BSI Surveillance systems description**																				
1	**1. Level of data aggregation for existing BSI epidemiological results available in the country**	1_1_Geographical aggregation (specify: national/regional/local)	**13**	X	X		X		X		X	X	X	X	X	X	X		X	X
	(Multiple selection)	1_2_Ward/specialty aggregation	**5**		X				X		X		X			X				
		1_3_hospital aggregation	**10**	X	X	X	X		X		X	X			X	X			X	
		1_4_Sex aggregation	**8**	X	X	X			X		X					X			X	X
		1_5_Age groups aggregation	**8**	X	X	X			X		X					X			X	X
		1_6_Hospital onset BSI or Health-associated BSI vs non HOB-BSI/non HA-BSI	**3**		X				X								X			
		1_7_Others	**2**					X		X										
1	**2. Mandatory/voluntary existent surveillance for BSI**	2_1_Mandatory for BSI attributed to some pathogens	**4**	X								X					X		X	
	(Single selection)	2_2_Mandatory for BSI attributed to any pathogen	**4**		X				X							X				X
		2_3_Voluntary for BSI attributed to some pathogens	**5**				X	X					X		X			X		
		2_4_Voluntary for BSI attributed to any pathogen	**4**			X				X	X			X						
		2_5_Non existent national surveillance for BSI	**0**																	
1	**3. Objective (s) of the current BSI surveillance system**	3_1_Participating in EARS net	**10**	X		X		X	X	X	X		X	X	X			X		
	(Multiple selection)	3_2_Participating in HAI-Net	**7**			X		X	X	X				X	X				X	
		3_3_HAI surveillance	**3**		X				X								X			
		3_4_BSI surveillance	**4**		X				X			X								X
		3_5_AMR surveillance	**9**			X		X	X		X		X	X	X	X			X	
		3_6_others	**1**								X									

II. Objectives, main characteristics, definitions and challenges of the EHR-BSI surveillance system																				
	**4. Type of source (s) of information to identify BSIs episodes for EHR-BSI surveillance**	4_1_Lab results	**16**		X	X	X	X	X	X	X	X	X	X	X	X	X	X	X	X
	(Multiple selection)	4_2_Hospital patient records	**4**			X						X							X	X
		4_3_Hospital discharge records	**1**																	X
		4_4_Others	**3**	X							X	X								
**2**	**5. Identify the different data sources that will be used for EHR-BSI surveillance**	5_1_Bloodstream infections or surveillance of invasive infections surveillance systems	**0**																	
	(Multiple selection)	5_2_Antimicrobial resistance surveillance systems	**3**								X		X					X		
		5_3_Notifiable disease surveillance systems	**0**																	
		5_4_Laboratory surveillance systems	**15**			X	X	X	X	X	X	X	X	X	X	X	X	X	X	X
		5_5_Hospital databases	**14**		X	X		X	X	X	X	X	X	X	X		X	X	X	X
		5_6_Primary care databases	**0**																	
		5_7_Pharmacy databases	**0**																	
		5_8_Insurance databases	**1**	X																
		5_9_Administrative databases	**3**						X				X						X	
		5_10_others	**4**								X						X		X	X
**2**	**6. Foreseen country coverage for EHR-BSI surveillance (population under surveillance) during the lifetime of the project (mid-2026)**	6_1_National	**9**	X	X				X	X					X	X	X	X		X
	(Single selection)	6_2_Selected Regions	**2**										X						X	
		6_3_Selected hospitals	**6**			X	X	X			X	X		X						
		6_4_Other	**0**																	
	**7. Objectives implementation: HA-BSI**	7_1_HA-BSI_1_Implemented	**2**						X							X				
	(Single selection)	7_1_HA-BSI_2_Under implementation	**11**		X	X		X		X	X	X	X	X			X		X	X
		7_1_HA-BSI_3_Planned during project	**4**	X			X								X			X		
		7_1_HA-BSI_4_No plans	**0**																	

	**7. Objectives implementation: Pandrug**	7_2_PDR_1_Implemented	**1**								X									
	(Single selection)	7_2_PDR_2_Under implementation	**5**							X		X	X		X					X
		7_2_PDR_3_Planned during project	**4**				X	X						X					X	
		7_2_PDR_4_Not implemented, no plans	**7**	X	X	X			X							X	X	X		
	**7. Objectives implementation: Emergent pathogens**	7_3_EmergentPathogens_1_Implemented	**1**								X									
	(Single selection)	7_3_EmergentPathogens_2_Under implementation	**3**										X		X					X
		7_3_EmergentPathogens_3_Planned during project	**7**			X	X	X		X		X		X					X	
		7_3_EmergentPathogens_4_Not implemented, no plans	**6**	X	X				X							X	X	X		
	**7. Objectives implementation: EARS-Net automatisation**	7_4_EARSAutom_1_Implemented	**1**								X									
	(Single selection)	7_4_EARSAutom_2_Under implementation	**6**					X		X			X		X			X		X
		7_4_EARSAutom_3_Planned during project	**1**									X								
		7_4_EARSAutom_4_Not implemented, no plans	**8**	X	X	X			X					X		X	X		X	
		7_4_EARSAutom_5_Unknown	**1**				X													
**2**	**8. How often you expect to collect and analyse the data by surveillance objective: HA-BSI**	8_1_HA-BSI_1_Yearly	**2**	X																X
	(Single selection)	8_1_HA-BSI_2_Quarterly	**9**		X	X		X		X		X		X	X			X	X	
		8_1_HA-BSI_3_Monthly	**0**																	
		8_1_HA-BSI_4_RealTime	**4**								X		X			X	X			
		8_1_HA-BSI_5_Other	**2**				X		X											
		8_1_HA-BSI_6_No	**0**																	
	**8. How often you expect to collect and analyse the data by surveillance objective: Pandrug**	8_2_Pandrug_1_Yearly	**1**								X									
	(Single selection)	8_2_Pandrug_2_Quarterly	**2**					X		X										
		8_2_Pandrug_3_Monthly	**0**																	
		8_2_Pandrug_4_RealTime	**6**				X					X	X	X	X					X
		8_2_Pandrug_5_Other	**1**																X	
		8_2_Pandrug_6_No	**7**	X	X	X			X							X	X	X		

	**8. How often you expect to collect and analyse the data by surveillance objective: Emergent Pathogens**	8_3_EmergentPathogens_1_Yearly	**1**								X									
	(Single selection)	8_3_EmergentPathogens_2_Quarterly	**2**			X				X										
		8_3_EmergentPathogens_3_Monthly	**0**																	
		8_3_EmergentPathogens_4_RealTime	**6**				X	X					X	X	X					X
		8_3_EmergentPathogens_5_Other	**1**																X	
		8_3_EmergentPathogens_6_No	**6**	X	X				X							X	X	X		
		8_3_EmergentPathogens_7_Unknown	**1**									X								
	**8. How often you expect to collect and analyse the data by surveillance objective: EARS-Net automatisation**	8_4_EARSAutom_1_Yearly	**2**								X									X
	(Single selection)	8_4_EARSAutom_2_Quarterly	**6**					X		X			X	X	X			X		
		8_4_EARSAutom_3_Monthly	**1**									X								
		8_4_EARSAutom_4_RealTime	**0**																	
		8_4_EARSAutom_5_Other	**1**				X													
		8_4_EARSAutom_6_No	**7**	X	X	X			X							X	X		X	
	**9. Can BSI episodes be distinguished for the EHR-BSI surveillance?** (Single selection)	9_1_Can BSI episodes be distinguished	**16**		X	X	X	X	X	X	X	X	X	X	X	X	X	X	X	X
**2**	**10. Minimum number of days between positive blood cultures with the same pathogen to be considered different BSI episodes in the EHR-BSI surveillance**	10_1_14 days	**12**		X	X	X	X		X	X	X			X	X	X	X		X
	(Single selection)	10_2_30 days	**4**						X				X	X					X	
		10_3_Differs	**0**																	
		10_4_Others	**1**	X																
**2**	**11. Causative pathogens planned to be under EHR-BSI surveillance**	11_1_Only EARS-net	**3**							X								X		X
	(Single selection)	11_2_All Bacteria	**1**										X							
		11_3_All pathogens	**11**	X	X	X	X	X	X		X	X		X	X	X				
		11_4_Selected list	**2**														X		X	

2	**12. Can healthcare-associated (HA)-BSI (available admission date +/- discharge date of previous healthcare admissions) be identified for the EHR-BSI surveillance?**	12_1_HA-BSI	**2**									X								X
	(Single selection)	12_2_HO-BSI	**7**		X	X		X			X			X		X			X	
		12_3_Both	**7**				X		X	X			X		X		X	X		
		12_4_None	**1**	X																
2	**13. Main challenge encountered**	16_1_Standarisation	**13**		X	X	X	X				X	X	X	X	X	X	X	X	X
	(Multiple selection)	16_2_Digitalisation	**6**	X		X	X	X				X							X	
		16_3_Data linkage technical	**7**	X		X	X	X			X					X				X
		16_4_Integration of systems	**7**	X	X			X				X	X	X						X
		16_5_Data protection issues	**6**	X			X						X			X	X	X		
		16_6_others	**10**		X	X		X	X	X				X	X	X		X	X	
**III. Readiness of national EHR systems in the perspective of EHR-BSI surveillance**																				
3	**14. Data stored in a structured way in EHR Systems**	17_1_Patient Characteristics_1_yes	**17**	X	X	X	X	X	X	X	X	X	X	X	X	X	X	X	X	X
	(Matrix)	17_1_Patient Characteristics_2_no	**0**																	
		17_1_Patient Characteristics_3_FreeText	**0**																	
		17_2_Patient origin transfer_1_yes	**12**			X	X	X	X	X	X		X		X	X	X		X	X
		17_2_Patient origin transfer_2_no	**5**	X	X							X		X				X		
		17_2_Patient origin transfer_3_FreeText	**0**																	
		17_3_Patient sign & symptoms_1_yes	**1**								X									
		17_3_Patient sign & symptoms_2_no	**7**	X			X					X			X		X	X		X
		17_3_Patient sign & symptoms_3_FreeText	**9**		X	X		X	X	X			X	X		X			X	
		17_4_Patient admissions & discharges_1_yes	**16**	X	X	X		X	X	X	X	X	X	X	X	X	X	X	X	X
		17_4_Patient admissions & discharges_2_no	**1**				X													
		17_4_Patient admissions & discharges_3_FreeText	**0**																	
		17_5_Patient main diagnosis codes_1_yes	**14**	X		X		X	X	X	X		X	X	X	X	X	X	X	X
		17_5_Patient main diagnosis codes_2_no	**3**		x		X					X								
		17_5_Patient main diagnosis codes_3_FreeText	**0**																	

		17_6_Hospital Unit, ward / speciality_1_yes	**16**	X	X	X	X	X	X	X	X	X	X	X	X	X	X		X	X
		17_6_Hospital Unit, ward / speciality_2_no	**1**															X		
		17_6_Hospital Unit, ward / speciality_3_FreeText	**0**																	
		17_7_Hospital type, primary, secondary, tertiary_1_yes	**16**	X	X	X	X	X	X	X		X	X	X	X	X	X	X	X	X
		17_7_Hospital type, primary, secondary, tertiary_2_no	**1**								X									
		17_7_Hospital type, primary, secondary, tertiary_3_FreeText	**0**																	
		17_8_Catheter procedures_1_yes	**5**				X		X	X	X								X	
		17_8_Catheter procedures_2_no	**7**	X								X			X	X	X	X		X
		17_8_Catheter procedures_3_FreeText	**5**		X	X		X					X	X						
		17_9_Origin of infection_1_yes	**2**								X								X	
		17_9_Origin of infection_2_no	**14**	X	X		X	X	X	X		X	X	X	X	X	X	X		X
		17_9_Origin of infection_3_FreeText	**1**			X														
		17_10_outcome death_1_yes	**11**		X			X	X	X	X			X		X	X	X	X	X
		17_10_outcome death_2_no	**5**	X			X					X	X		X					
		17_10_outcome death_3_FreeText	**1**			X														
		17_11_Blood culture performed & results_1_yes	**14**		X	X		X	X	X	X	X	X	X	X	X	X		X	X
		17_11_Blood culture performed & results_2_no	**3**	X			X											X		
		17_11_Blood culture performed & results_3_FreeText	**0**																	
		17_12_Susceptibility tests performed & AST results_1_yes	**14**		X	X		X	X	X	X	X	X	X	X	X	X		X	X
		17_12_Susceptibility tests performed & AST results_2_no	**3**	X			X											X		
		17_12_Susceptibility tests performed & AST results_3_FreeText	**0**																	
		17_13_Denominators_1_yes	**14**	X	X	X	X	X	X		X	X	X	X	X		X		X	X
		17_13_Denominators_2_no	**3**							X						X		X		
		17_13_Denominators_3_FreeText	**0**																	

	**15. Data extracted using national or regional protocols**	18_1_Patient Characteristics	**13**	X	X	X		X	X	X	X		X		X		X	X	X	X
	(Matrix)	18_2_Patient origin transfer	**8**			X		X	X	X					X		X		X	X
		18_3_Patient sign & symptoms	**0**																	
		18_4_Patient admissions & discharges	**14**	X	X	X		X	X	X	X		X		X	X	X	X	X	X
		18_5_Patient main diagnosis codes	**11**	X	X	X		X	X	X			X		X		X		X	X
		18_6_Hospital Unit, ward / speciality	**12**	X	X			X	X	X	X		X		X	X	X		X	X
		18_7_Hospital type primary, secondary, tertiary	**14**	X	X	X		X	X	X	X		X		X	X	X	X	X	X
		18_8_Catheter procedures	**3**						X	X									X	
		18_9_Origin of infection	**1**																X	
		18_10_outcome death	**6**						X	X							X	X	X	X
		18_11_Blood culture performed & results	**14**		X	X		X	X	X	X	X	X		X	X	X	X	X	X
		18_12_Susceptibility tests performed & AST results	**14**		X	X		X	X	X	X	X	X		X	X	X	X	X	X
		18_13_Denominators	**11**	X	X			X	X		X		X		X		X	X	X	X
**IV. Data processing and standardisation**																				
**4**	**16. Expected level of automation on the EHR-BSI Surveillance System during the lifetime of the project (mid-2026)**	13_1_Automatic data extraction/data collection	**17**	X	X	X	X	X	X	X	X	X	X	X	X	X	X	X	X	X
	(Multiple selection)	13_2_Automatic data linkage/merging datasets	**11**			X			X	X	X	X	X	X	X		X	X	X	
		13_3_Automatic case phenotyping	**11**		X				X	X	X	X		X	X		X	X	X	X
		13_4_Automatically identifying two cultures with skin contaminants	**6**						X		X			X	X				X	X
		13_5_Automatic triggering of alerts within the surveillance/monitoring system	**2**						X						X					
		13_6_Automatic data exchange / reporting	**8**	X	X				X		X	X			X		X		X	
		13_7_Automatic denominator calculation	**6**					X	X		X				X		X		X	
**4**	**17. Specify if manual data validation is necessary**	14_1_Already validated	**1**						X											
	(Single selection)	14_2_Well-defined but manual	**4**		X					X	X							X		
		14_3_Planned to be defined	**12**	X		X	X	X				X	X	X	X	X	X		X	X
		14_1_No plans	**0**																	

4	**18. IT tool planned to be used to map & extract data from EHR systems**	19_1_Tools are yet to be chosen	**7**	X	X		X	X				X	X							X
	(Multiple selection)	19_2_FHIR profiles (or equivalent) will be used to describe for EHR sources	**0**																	
		19_3_A FHIR API (or equivalent) will be implemented by Hospital / Labs	**5**			X				X	X					X		X		
		19_4_A central API will be used to allow sites to submit data on a given format	**7**						X		X			X			X	X	X	X
		19_5_A given data extraction software will be installed on Hospital / Labs to collect data	**1**												X					
		19_6_A secured file server will be used to submit data to a central level	**1**		X															
	**19. Data model used/planned in existing national or regional level databases collecting data from EHR**	20_1_National developed schema	**15**	X	X		X	X	X	X	X		X	X	X	X	X	X	X	X
	(Multiple selection)	20_2_Regional developed schema	**0**																	
		20_3_OMOP	**2**			X										X				
		20_4_Other	**0**																	
		20_5_Not available	**1**									X								
5	**20. Standard vocabularies / terminologies planned to be used to standardise EHR based data**	21_1_Patient variables_1_SNOMED	**3**		X			X												X
	(Multiple selection)	21_1_Patient variables_2_National	**1**								X									
		21_1_Patient variables_3_Unknown	**8**	X			X					X	X		X	X	X	X		
		21_1_Patient variables_4_Other	**5**			X			X	X				X					X	
		21_2_Microorganisms_1_SNOMED	**8**	X	X	X		X		X	X					X			X	
		21_2_Microorganisms_2_LOINC	**4**			X					X		X	X						
		21_2_Microorganisms_3_WHOCARE	**4**					X				X			X					X
		21_2_Microorganisms_4_National	**1**											X						
		21_2_Microorganisms_5_NCBI	**1**															X		
		21_2_Microorganisms_6_NPU	**1**														X			
		21_2_Microorganisms_7_EARS-Net	**0**																	
		21_2_Microorganisms_8_Unknown	**1**				X													
		21_2_Microorganisms_9_Other	**1**						X											

		21_3_Cathether procedures_1_SNOMED	**2**		X						X									
		21_3_Cathether procedures_2_National	**1**																X	
		21_3_Cathether procedures_3_Unknown	**12**	X		X	X	X				X	X	X	X	X	X	X		X
		21_3_Cathether procedures_Other	**2**						X	X										
		21_4_Diagnostics_1_ICD9	**1**										X							
		21_4_Diagnostics_2_ICD10	**8**	X		X			X	X					X		X	X		X
		21_4_Diagnostics_3_ICD11	**3**					X		X				X						
		21_4_Diagnostics_4_SNOMED	**3**		X			X			X									
		21_4_Diagnostics_5_Unknown	**3**				X					X				X				
		21_4_Diagnostics_6_Other	**1**																X	
		21_5_Antimicrobials_1_LOINC	**3**		X					X	X									
		21_5_Antimicrobials_2_WHOCARE	**2**										X		X					
		21_5_Antimicrobials_3_NPU	**1**														X			
		21_5_Antimicrobials_4_EARSNet	**4**					X								X		X	X	
		21_5_Antimicrobials_5_ATC	**3**			X							X	X						
		21_5_Antimicrobials_6_Unknown	**4**	X			X					X								X
		21_5_Antimicrobials_7_Other	**2**					X	X											
**V. Patient linkage between EHR Systems**																				
	**21. Patient unique identifier (ID) for data linkage between EHR-BSI data sources.**	22_1_National unique ID at hospital level	**0**																	
	(Multiple selection)	22_2_Unique patient ID at the hospital level	**1**															X		
		22_3_Unique patient ID at the Lab level	**3**				X									X		X		
		22_4_Unique patient ID linkable with Lab and clinical data	**14**	X	X	X		X	X	X	X	X	X	X	X		X		X	X
		22_5_Unique ID not available	**0**																	
		22_6_Unique ID planned to be constructed during the lifespan of the project	**0**																	

4	**22. Is data linkage between hospital, lab and other data sources possible for EHR-BSI surveillance?**	23_1_Linkage_1_Yes	**11**		X	X		X	X	X	X	X		X	X			X	X	
	(Single selection)	23_1_Linkage_2_Partially	**1**										X							
		23_1_Linkage_3_No by law(GDPR)	**1**														X			
		23_1_Linkage_4_No technical	**2**				X													X
		23_1_Linkage_4_No both	**2**	X												X				
		23_1_Linkage_6_No other	**0**																	
4	**23. Level of data linkage: at what level would data be linked in the foreseen EHR BSI surveillance system?**	24_1_EHRHOSP local level	**5**		X	X		X			X			X						
	(Multiple selection)	24_2_EHRREG regional level	**2**										X						X	
		24_3_EHRNAT national level	**9**			X			X	X		X			X		X	X	X	X
		24_4_No linkage	**3**	X			X									X				
		24_5_Unknown	**0**																	
**VI. Denominators**																				
	**24. Planned level of data aggregation for EHR-BSI results**	25_1_Geographical aggregation	**14**		X	X	X	X	X	X			X	X	X	X	X	X	X	X
	(Multiple selection)	25_2_Ward/specialty	**12**		X	X	X	X	X			X	X	X	X	X			X	X
		25_3_hospital or type of hospital	**14**	X	X		X	X	X	X		X	X	X	X	X		X	X	X
		25_4_Sex aggregation	**11**	X		X		X	X			X	X	X	X	X			X	X
		25_5_Age groups aggregation	**11**	X		X		X	X			X	X	X	X	X			X	X
		25_6_Hospital onset BSI or Health-associated BSI vs non HOB-BSI/non HA-BSI	**9**				X	X					X		X	X	X	X	X	X
		25_7_ all	**4**								X		X		X					X
		25_8_Others	**2**	X					X											
2	**25. Denominators available to calculate BSI incidence for the EHR-BSI surveillance**	26_1_Patient-days	**14**		X	X	X	X	X	X	X	X	X	X	X		X	X		X
	(Multiple selection)	26_2_Discharges/admissions	**7**		X					X		X	X		X	X				X
		26_3_Catchment Population	**8**	X	X	X				X			X	X	X			X		
		26_4_All	**4**		X								X		X				X	
		26_5_None	**0**																	

	**26. Is there data available on the number of blood culture sets?**	27_1_Available by patient days	**11**		X	X	X	X	X			X	X	X	X				X	X
	(Single selection)	27_2_Available by population	**2**															X	X	
		27_3_Not available	**5**	X						X	X					X	X			
**2**	**27. Are denominators available by ward / speciality?**	28_1_Yes	**10**				X	X	X		X	X		X	X		X		X	X
	(Single selection)	28_2_Yes, ICU / non-ICU	**2**		X	X														
		28_3_No	**4**	X						X						X		X		
		28_4_Unknown	**1**										X							
**VII. Legal issues reported by countries**																				
	**28. Are there legal issues preventing the collection of data at National level for EHR-BSI surveillance?**	15_1_Case level data cannot be used for surveillance at national level	**1**															X		
	(Multiple selection)	15_2_Patient hospital data cannot be linked to use for surveillance at national level	**3**										X			X	X			
		15_3_Lab data cannot be linked to use for surveillance at national level	**2**										X				X			
		15_4_Other limitations	**1**	X																
		15_5_No limitations	**12**		X	X	X	X	X	X	X	X		X	X				X	X
	**29. Are there particular regulatory limitations for sharing aggregated results within the EHR-BSI project?**	29_1_Yes	**0**																	
	(Single selection)	29_2_Exploring	**7**	X			X	X	X			X						X		X
		29_3_No	**10**		X	X				X	X		X	X	X	X	X		X	
	**30. Are there particular regulatory limitations for sharing the pseudonymised case-based data indicated in the protocol with ECDC?**	30_1_Yes	**4**						X				X			X	X			
	(Single selection)	30_2_Exploring	**7**	X		X	X	X				X						X		X
		30_3_No	**6**		X					X	X			X	X				X	

**VIII. Data Sharing**																				
	**31. When will you be able to provide data during the timeframe of the project?**	31_1_2nd Q 2024	**0**																	
	(Single selection)	31_2_3rd Q 2024	**0**																	
		31_3_4th Q 2024	**0**																	
		31_4_1st Q 2025	**1**							X										
		31_5_2nd Q 2025	**5**			X					X	X			X		X			
		31_7_3rd Q 2025	**1**													X				
		31_7_4th Q 2025	**5**					X	X				X	X						X
		31_8_1st Q 2026	**5**	X	X		X											X	X	
		31_9_No plans	**0**																	
4	**32. In which granularity will you share data?**	32_1_Aggregated	**7**				X		X			X	X			X	X		X	
	(Single selection)	32_2_Case based	**3**								X			X	X					
		32_3_both	**5**		X	X		X		X										X
		32_4_Not decided	**2**	X														X		
		32_5_ No plans	**0**																	

Electronic Health Record (EHR)-based Bloodstream Infections (BSI) Generic protocol – abridged version v0.3 (2 October 2024)

1. Description of the surveillance system 2
1.1. Aim and objectives 2
1.1.1. Overall aim – Justification of implementation 2
1.1.2. Surveillance objectives 2
1.2. Study population 3
1.3. Data collection period 3
1.4. Key definitions 4
1.4.1. Laboratory-confirmed Bloodstream infection (BSI) case definition 4
1.4.2. Contextual BSI data 4
1.4.3. Microorganisms under surveillance 5
1.4.4. Personal identifiers and data linkage 5
1.4.5. Episode definitions 6
1.4.6. Healthcare-associated, hospital-onset Healthcare-associated, and community-associated BSIs 7
1.5. Variables to be collected 7
1.6. Granularity of data collected 10
1.7. Data processing, interoperability and structure 10
2. Data management and analysis 12
2.1. Type of analysis 12
2.2. Data extraction 12
2.3. Denominator 12
2.4. Censoring events (only in case-based analysis) 13
2.5. Data checking and validation 13

1. Description of the surveillance system

1.1. Aim and objectives

1.1.1. Overall aim – Justification of implementation

This project aims to support participating countries to develop and expand EHR based surveillance systems for BSI within a harmonised EU/EEA surveillance system to inform public health action for disease prevention and control at local, national and European levels by:

- Creating a European network which will facilitate building national and European BSI surveillance systems based on EHR.
- Building a system to enable reporting of an individual's episodes of care (e.g., admissions to different hospitals, readmission with BSI, etc).
- Creating a database linking patient and pathogen information.
- Digitalising and automatisation of BSI surveillance to reduce workloads and improve timeliness, which may result in an improved system that can provide information about a possible increase in cases or a certain resistance pattern. Collecting data on emerging or re-emerging pathogens causing BSI (e.g., outbreaks of *C. auris*).
- Automatically collecting standardised data on AMR profiles, including pan-drug resistance, of BSI isolates.

1.1.2. Surveillance objectives

Through routinely collected data from EHR, and by automatising/semi-automatising as much as possible specific processes within the surveillance systems, the project surveillance objectives include:

1) Incidence of healthcare-associated (HA) BSI
2) Possible pan-drug resistance (PDR) in BSIs
3) Emerging pathogens in BSIs (e.g., *C. auris*)
4) AMR data according to EARS-Net protocol

Primary and secondary surveillance objectives

We consider as the primary surveillance objective the establishment of automated EHR based surveillance of BSI for the estimation of HA-BSI incidence by collecting data from EHR (objective 1). A better and more timely understanding of the most frequent microorganisms causing BSI, including AMR profiles, by country or region, sites (hospitals and wards), subpopulations (e.g., age groups) and risk groups (e.g. procedures) will enable more timely prevention and control actions.

We consider the secondary objectives to be:

- Establishing the surveillance and alert of
  - possible pan-drug resistant microorganisms
  - emerging pathogens

  In these cases, it is crucial to have the information as close to real time as possible in order to control outbreaks or the spread of a specific resistant microorganism.
- Achieving full digitalisation and reducing manual steps in the EARS-Net reporting process. This last objective will reduce workload for countries to report to ECDC and so facilitate increased frequency from the current yearly reporting.

Countries can prioritise which of the surveillance objectives to address according to national context and needs. Implementing all the different surveillance objectives in each country is a longer-term process and each one of the objectives can be achieved gradually over time. For this reason, we envisage that some

countries will prefer to address one or more of the secondary surveillance objectives first and not the primary surveillance objective of estimating BSI incidence. For example, countries could start using an isolate-based system with limited data linkage between laboratory data and patient and clinical specific variables. This may be the case of the surveillance and alert of possible pan-drug resistant and emerging pathogens as well as the automatisation of the EARS-Net protocol (objectives 2 to 4), which, as an initial step, can be more easily achieved by starting with an isolate-based system.

1.2. Study population

The primary objective of the protocol is to measure HA-BSI incidence, so the study population will be all hospitalised patients within the study site, whether that be a single hospital, a group of hospitals or within a geographic area (district, region or national).

A hospitalised patient is defined as someone admitted for a defined period of time, whether this is calculated as the number of hours (e.g., >24 hours) or days (e.g., an admission of >1 day).

When possible, data from all cases of BSI, HA-BSI or hospital-onset HA-BSI and community-associated BSI (CA-BSI) from the whole country will be included.

1.3. Data collection period

The project aims to collect and report data timely enough to allow actions for disease prevention and control. This is understood as producing a real-time single data set from which all the objectives of the project can be achieved (see table 1).

But taking into account the challenge of digitalisation and automatisation for many countries, initially countries can aim to collect and report data differently for each project objective.

For objectives 1 (HA-BSI incidence) and 4 (EARS-Net reporting), countries should aim to collect and report all data sources quarterly or more frequently (see table 1). Although less frequent reporting can be accepted (e.g., biannual or annual) at the start of the project. For objectives 2 (BSI PDR) and 3 (Emerging BSI pathogens), countries should aim to provide data as close to real-time as possible (see Table 1).

Table 1. Suggested data collection periods by objectives.

Objective 1. Healthcare-associated BSI (HA-BSI incidence)	Quarterly
Objective 2. Possible pan-drug resistance in BSIs (PDR) (surveillance and alert)	Near real-time
Objective 3. Emerging pathogens in BSIs (surveillance and alert)	Near real-time
Objective 4. AMR according to EARS-Net protocol	Quarterly

1.4. Key definitions

In this updated protocol, we have endeavoured to ensure as much as possible that the definitions employed are aligned with those used by both the PRAISE network and the TESSy Reporting Protocol for EHR v0.1.

1.4.1. Laboratory-confirmed Bloodstream infection (BSI) case definition

The ECDC case definition used is coherent with the one published in the Official Journal of the European Union (Commission Implementing Decision (EU) 2018/945)[1]:

- Patient has at least one positive blood culture for a recognised pathogen

OR

- two positive blood cultures for the same species/subtype of common skin contaminant (from two separate blood cultures, within three calendar days) (the first date of specimen = day one)

*Common skin contaminants/Common commensals: The European definition lists five possible common skin contaminants (*coagulase-negative staphylococci, Micrococcus spp., Propionibacterium acnes, Bacillus spp., Corynebacterium spp*.). However, a more complete list is provided by the CDC's National Healthcare Safety Network (NHSN) Common Commensals microorganism list, version February 2024[2].

In the ECDC official EU case definition, for a common skin contaminant identified in blood, it is also required that the patient has at least one of the following signs or symptoms: fever (> 38 °C), chills, or hypotension. However, several countries have pointed out that clinical symptoms are usually either not recorded or recorded as free text in EHR complicating collection of these data in an automated way, and therefore the definition above omits the symptoms. When symptoms cannot be collected, the case definition for common skin contaminants may be two separate positive blood cultures with the same pathogen as outlined above. Furthermore, some countries may be able to timestamp their samples, in which case "within three calendar days" can be replaced by "within 48 hours'' as published in the EU case definition.

1.4.2. Contextual BSI data

To identify BSI cases, a dataset with results from all positive blood cultures performed during each study period for the defined area under surveillance will be required. Data about the causative pathogen will be collected. If possible, the total number of blood cultures performed will also be reported in aggregated format (see variable "Number of blood culture sets per year" from the TESSy Reporting Protocol for EHR-BSI v0.1).

---

[1] European Centre for Disease Prevention and Control. EU case definition, webpage last updated 3 Jul 2018. https://www.ecdc.europa.eu/en/all-topics/eu-case-definitions

[2] CDC, Organisms categorized as Common Commensals, to be used for all organisms identified January 1, 2024 through December 31, 2024 (updated 02-2024): https://view.officeapps.live.com/op/view.aspx?src=https%3A%2F%2Fwww.cdc.gov%2Fnhsn%2Fxls%2Fmaster-organism-com-commensals-lists.xlsx&wdOrigin=BROWSELINK

1.4.3. Microorganisms under surveillance

We recommend monitoring all microorganisms detected in blood cultures. However, depending on data availability and national priorities, countries may prefer to start implementing the protocol focusing on:

- **Microorganisms under surveillance in EARS-Net**
  In this category we include the eight microorganisms currently under surveillance in EARS-Net: *Streptococcus pneumoniae*, *Staphylococcus aureus, Enterococcus faecalis, Enterococcus faecium, Escherichia coli, Klebsiella pneumoniae, Pseudomonas aeruginosa, Acinetobacter spp*[3]

In addition, countries may select any of the microorganism groups listed below:

- **Common commensals/skin contaminants** (see section 1.4.1 Laboratory-confirmed BSI case definition)
- **Fungi**
- **Other microorganisms**
  Any other microorganisms not covered in the above categories, including emerging microorganisms (any pathogen considered emerging or re-emerging in the country).

Countries need to provide the list of microorganisms under surveillance

We envisage that at the start of the project, countries may prefer to work with their own list/categories of common commensals/skin contaminants. For example, countries may wish to include only coagulase-negative staphylococci and exclude other skin contaminants. If this is the case, they will need to provide their own list of microorganisms under surveillance. Furthermore, it is required that countries share classification system in use for microbiological data in EHRs (see variable "MicrobiologicalTerminology" in the TESSy Reporting Protocol for EHR-BSI v0.1)

Besides providing data on the pathogens to be monitored, when possible, hospital admission codes (e.g., International Statistical Classification of Diseases and Related Health Problems 10th Revision -ICD-10- or 11 Revision -ICD-11) related to BSI (infection) should be collected.

1.4.4. Personal identifiers and data linkage

<u>Personal identifiers</u>

To identify individuals and episodes of care, a personal identifier (ID) is needed to link records from different data sources (patient transfers) or to deduplicate records from the same episode of care (e.g., multiple blood cultures, see section 1.4.5). If available in the country, a unique personal identifier will be used, such as a pseudonymised national insurance number.

Other countries may use unique hospitalisation codes (patient's Hospital IDs) which may change for the same individual in different hospitals. The patient's hospital ID can be theoretically linked to a national ID but, even if this is not possible, it can be used to discriminate between episodes that have occurred in the same hospital. On some occasions, a patient's laboratory level ID (patient's Lab IDs) is necessary to link a patient's hospital ID to the associated diagnosis of the patient.

---

[3] WHO Regional Office for Europe/European Centre for Disease Prevention and Control. Antimicrobial resistance surveillance in Europe 2022 – 2020 data. Copenhagen: WHO Regional Office for Europe; 2022.

All data submitted will be processed conforming to national and European data protection regulations. The data submitted to ECDC will include a pseudo-anonymised identifier that allows the reporting countries/sites to go back and possibly ask for clarifications regarding a particular case.

Data linkage

If a unique personal identifier is not available, countries will describe which variables can be used for linkage and the methods they can use to link different datasets and registries in their country to collect information for each individual case.

These are the main methods of data linkage and their characteristics[4] are:

- *Deterministic linkage:* pairs of records are classified as matches if their linking variables predominantly agree.
- *Probabilistic linkage:* record pairs are given scores representing the likelihoods of belonging to the same individual given the strength of agreement of variables (i.e. the strength of the match between record pairs). This method may be applied where there are no unique identifiers or linking keys, where linking variables are not reliable[5].

1.4.5. Episode definitions

Episodes and potential related variables need to be computed but are not requested in the TESSy Reporting Protocol for EHR-BSI v0.1.

The date of onset of the episode will be the date of specimen collection in specimen with pathogen or first date of specimen with common skin contaminant that is a part of a set of at least two cultures (see variable "DateOfSpecCollection" in the ECDC Reporting Protocol for EHR-BSI v0.1). If not available, the following dates can be used as date of onset in the order presented here: i) date of onset of signs or symptoms coherent with a BSI, ii) date of diagnosis, or iii) date of notification (understood as reporting date).

BSI episode

An episode of BSI is defined for each BSI matching the case definition as a 14-day period defined starting from the date of onset (date of onset = day one). In case the same species/subtype is reported multiple times within the 14-day episode, these recurring isolates are considered to be part of the same episode.

We have used a period of 14 days to define an episode to be consistent with both the PRAISE[6]network and the TESSy Reporting Protocol for EHR-BSI v0.1. However, the period defining episodes may vary by country if different period duration (e.g. 30-days) has been used, and this may be reported in the TESSy Reporting Protocol v0.1[7-8].

---

[4] European Union, EUROSTAT, Collaboration in Research and Methodology for Official Statistics (CROS), Methodology data linkage, webpage last updated 5 Jul 2023. https://cros-legacy.ec.europa.eu/content/s-dwh-m42methodologydatalinkagev2pdf_en

[5] Potz, Nicola, Powell, David, Lamagni, Theresa L, et al. "Probabilistic Record Linkage of Infection Records and Death Registrations: A Tool to Strengthen Surveillance" Statistical Communications in Infectious Diseases, vol. 2, no. 1, 2010. https://doi.org/10.2202/1948-4690.1015

[6] Seven J.S. Aghdassi, Suzanne D. van der Werff, Gaud Catho, et al. Hospital-onset bacteraemia and fungaemia as a novel automated surveillance indicator: results from four European university hospitals. medRxiv 2024.09.16.24310433; doi: https://doi.org/10.1101/2024.09.16.24310433

[7] Holmbom M, Giske CG, Fredrikson M, et al. 14-Year Survey in a Swedish County Reveals a Pronounced Increase in Bloodstream Infections (BSI). Comorbidity - An Independent Risk Factor for Both BSI and Mortality. PLoS One. 2016 Nov 11;11(11):e0166527. doi: 10.1371/journal.pone.0166527. PMID: 27835663; PMCID: PMC5106013.

[8] Kontula KSK, Skogberg K, Ollgren J, et al. Population-Based Study of Bloodstream Infection Incidence and Mortality Rates, Finland, 2004-2018. Emerg Infect Dis. 2021 Oct;27(10):2560–9. doi: 10.3201/eid2710.204826. PMID: 34546161; PMCID: PMC8462341

Episode type (Monomicrobial/Polymicrobial episodes)

The BSI can be:

- Monomicrobial episode: episode in which only one species/subtype is isolated within three calendar days from the date of onset (from the first positive blood culture).
- Polymicrobial episode: episode in which more than one species/subtype are isolated within three calendar days from the date of onset (from the first positive blood culture). In case of reporting of different species/subtype after three or more calendar days after the date of onset, a new 14-day BSI episode is recorded.

1.4.6. Healthcare-associated, hospital-onset Healthcare-associated, and community-associated BSIs

We envisage that in most countries, the blood cultures will be almost exclusively taken at hospitals providing secondary and tertiary healthcare, at emergency departments, inpatient wards or (rarely) in hospital-based outpatient care. We recommend that dates and times of admission, discharge and specimen collection should be collected when available, allowing for healthcare-associated or community-associated episodes to be reported.

Episode definitions for healthcare-associated (HA)-BSI, hospital-onset HA-BSI and imported HA-BSI

A BSI episode is defined as HA BSI episode if the date of onset is on day 3 or later of the hospital admission (date of admission = day one) or within three calendar days after a discharge from a healthcare facility (date of discharge = day one).

- A BSI episode is defined as a Hospital-onset HA-BSI episode if the date of onset is on day 3 or later of the hospital admission (date of admission = day one).
- A BSI episode is defined as an Imported HA-BSI episode, i.e. episodes with readmission for BSI, if the date of onset is within three calendar days after a discharge from a healthcare facility (date of discharge = day one).

Community-associated (CA) episode

Those BSI episodes in non-hospitalised patients or patients hospitalised (but not transferred from another healthcare facility) with date of onset before day 3 of hospitalisation (date of admission = day one).

Total BSI episodes

All recorded BSI episodes

1.5. Variables to be collected

The number and types of variables to be collected, as outlined in this protocol, has been reduced compared to previous versions and has been aligned more closely to the structure proposed in the "TESSy Reporting Protocol for EHR-BSI, version 0.1". Nonetheless, this protocol does list a larger number of variables than that proposed in the TESSy Reporting Protocol as the intention is to anticipate future needs and developments of the EHR surveillance system.

The TESSy Reporting Protocol for EHR-BSI v0.1 is accompanied by a metadata set *"to support the timely and complete reporting of key information for surveillance of bloodstream infections at local, regional/national and European level by providing flexible options for uploading aggregated or case-based EHR-BSI data to ECDC".* In the reporting protocol, the variables have been divided into five datasets in hierarchical levels, which can be divided into aggregated and case-based reporting sets (case-based reporting includes aggregated denominators and other items). The $-sign below indicates the hierarchical

relations between the levels, and the relation between levels is indicated with RecordId-ParentId-pairs. These are the five datasets and their variables:

1. Surveillance type and hospital-related dataset (named as ´EHRBSI´ level of reporting in the metadata): ´EHRBSI´ level is mandatory to be used for all reporting of EHRBSI data, case-based as well as aggregated. This level includes variables from three different groups:
    a. Common TESSy variables (referring to the structure of the relational CSV/XML files)
        i. Record Identifier (mandatory)
        ii. Record type (mandatory)
        iii. Record type version
        iv. Subject (mandatory)
        v. Status (mandatory)
        vi. Data source (mandatory)
        vii. Reporting country (mandatory)
        viii. Date used for statistics (mandatory)
    b. Variables on hospital and surveillance system characteristics
        i. Hospital identifier
        ii. Laboratory code
        iii. Geographical location
        iv. Hospital size
        v. Hospital type
        vi. Current degree of automation of surveillance of HA-BSI
        vii. Level of data aggregation
        viii. Definition of duration of BSI episode
        ix. Terminology / classification system in use for clinical data in EHRs
        x. Specification of the terminology / classification system in use for clinical data in EHRs
        xi. Terminology / classification system in use for microbiological data in EHRs
        xii. Specification of the terminology / classification system in use for microbiological data in EHRs
    c. Variables on high-level aggregated indicators and denominators
        i. Number of blood culture sets
        ii. Number of discharges (or admissions) per surveillance period
        iii. Number of patient-days per surveillance period
        iv. Estimated proportion of the national or regional population covered by the surveillance
        v. Number of hospital-onset HA-BSIs
        vi. Number of imported HA-BSIs
        vii. Number of total BSIs

2. Denominator dataset (aggregated and case-based data) (named as "EHRBSI$Denom" level of reporting in the metadata): ´EHRBSI$Denom´ is optional and may be used together with ´EHRBSI' if reporting data at hospital unit level. This level includes variables from two different groups:
    a. Common TESSy variables (referring to the ´EHRBSI´ tables)
        i. Record Identifier (mandatory)
        ii. Parent Identifier (mandatory, linking to EHRBSI RecordID)
    b. Variables for stratified aggregated indicators and denominators
        i. End date of this surveillance period (mandatory)
        ii. Start date of this surveillance period (mandatory)
        iii. Unit Identifier
        iv. Specialty of the Unit (Ward)
        v. Number of discharges (or admissions) per surveillance period per selected stratification
        vi. Number of patient-days per surveillance period per selected stratification
        vii. Number of hospital-onset HA-BSIs per selected stratification
        viii. Number of imported HA-BSIs per selected stratification

    ix. Number of total BSIs per selected stratification

3. Information on patient’s dataset (case-based data) (named as “EHRBSI$Patient” level of reporting in the metadata): ´EHRBSI$Patient´ is optional and may be used together with ´EHRBSI’ if reporting case-based data on BSIs and is used to report information related to the patient. This level includes variables from two different groups:
    a. Common TESSy variables (referring to the ´EHRBSI´ table)
        i. Record Identifier (mandatory)
        ii. Parent Identifier (mandatory, linking to EHRBSI RecordID)
    b. Variables for patient information
        i. Unit Identifier (the unit where the sample was taken according to the date of specimen collection)
        ii. Specialty of the Unit (Ward)
        iii. Consultant/Patient specialty
        iv. Date Of Admission Current Ward
        v. Patient counter
        vi. Age
        vii. Gender
        viii. Origin of patient
        ix. Date of hospital admission
        x. Date of hospital discharge
        xi. Outcome
        xii. Primary code for hospital discharge or admission
        xiii. Primary code label of admission event of the patient
        xiv. Code system of the primary code of the admission event of the patient
        xv. Specification of the code system of the primary code of the admission event of the patient
        xvi. Code system version of the primary code of the admission event of the patient
        xvii. Previous admission to a healthcare facility

4. Isolate-based dataset (isolate-based data) (named as “EHRBSI$Patient$Isolate” level of reporting in the metadata): ´EHRBSI$Patient$Isolate’ is optional and may be used together with ´EHRBSI’ and ´EHRBSI$Patient´ if reporting case-based data on BSIs and is used to report information related to the microbiological sample/isolate. This level includes variables from two different groups:
    a. Common TESSy variables (referring to the EHRBSI$Patient)
        i. Record Identifier (mandatory)
        ii. Parent Identifier (mandatory, linking to EHRBSI$Patient RecordID)
    b. Variables for the information on the isolate
        i. Date of specimen collection (mandatory)
        ii. Laboratory code
        iii. Isolate Identifier
        iv. Code of the microorganism responsible for the BSI episode
        v. Code label of the microorganism responsible for the BSI episode
        vi. Code system that includes the code of the microorganism responsible for the BSI episode
        vii. Specification of the code system that includes the code of the microorganism responsible for the BSI episode
        viii. Code system version that includes the code of the microorganism responsible for the BSI episode

5. AMR profiles of isolates dataset (isolate-based data) (named as “EHRBSI$Patient$Isolate$Res” level of reporting in the metadata): ´EHRBSI$Patient$Isolate$Res’ is optional and may be used together with ´EHRBSI’, ´EHRBSI$Patient´ and ´EHRBSI$Patient$Isolate´ if reporting case-based data on BSIs including antimicrobial resistance results. This level is used to report information related to the antimicrobial susceptibility testing of the microbiological sample/isolate. The level

and all variables are optional, also for case-based reporting. This level includes variables from two different groups:

a. Common TESSy variables (referring to the ´EHRBSI$Patient$Isolate table
    i. Record Identifier (mandatory)
    ii. Parent Identifier (mandatory, linking to EHRBSI$Patient$Isolate RecordID)
b. Variables for the results on the antimicrobial susceptibility testing
    i. Antibiotic code
    ii. SIR
    iii. PCR mec-gene
    iv. PBP2a-agglutination
    v. ESBL present
    vi. Carbapenemases
    vii. Zone value
    viii. Interpretation of zone test
    ix. MIC sign
    x. MIC value
    xi. Interpretation of MIC test
    xii. Gradient strip sign
    xiii. Gradient strip value
    xiv. Interpretation of the gradient strip test
    xv. Disk load
    xvi. Reference Guidelines SIR

Under the mandatory variable to be reported ´data source´, within the "Surveillance type and hospital-related dataset", the investigators should identify which data source the variable (or group of variables) will be collected from. In cases where a variable is available in two or more data sources, hierarchical rules should be included to decide which data source the variable will be collected from in cases of divergent or missing information.

1.6. Granularity of data collected

It is recommended to report case-based data by hospital and with specialty-specific aggregation of denominator data to allow for stratified epidemiological analysis.

BSI data can be reported in both formats, aggregated or case-based, to achieve the primary surveillance objective of estimating the incidence of HA-BSI. This allows those countries that cannot report case-based data to report aggregated data on BSIs at the national, regional, hospital or laboratory level.

1.7. Data processing, interoperability and structure

The protocol proposes a BSI EHR-based surveillance system which defines data processing from laboratories and hospitals (and other data sources) into a centralised database to compute indicators for country and EU-wide BSI surveillance. Different countries may choose different data processing depending on the particularities of their data sources and existing surveillance systems.

There are different possible models for organising data flows that could suit different scenarios. However, the recommended option is a model where data storage is at the national level and processes such as standardisation, validation and calculation of indicators can be carried out at any level (local, regional, national or EU) according to national needs.

Pseudonymisation approaches produced by non-reversible hashing techniques are recommended to ensure that, at least at the hospital/lab level, each individual is associated with a unique and stable identifier. Data will be submitted to ECDC through TESSy/EpiPulse as described in the Reporting Protocol

for EHR-BSI v0.1. The coordination team, with the support of ECDC, will engage with individual countries to define the most suitable approach for data submission taking in consideration possible GDPR constraints.

Some variables (i.e. treatments, pathogens, diagnoses and procedures) will need to be coded using standardised vocabularies. A country specific codification system will be considered compatible if a mapping towards the target vocabulary exists or if it is expected to be developed in the near future. The mapping implementation can be done in iterations, including on the first steps only a subset of codes and defining a roadmap for the mapping of the pending codes. The coordination team will advise and support countries and pilots to choose the best strategy to select and map standards to use.

It is recommended to use the proposed data structure for case-based and aggregated, at local, regional or national level even in cases when only aggregated data is submitted to TESSy. We propose the tabular structure (Figure S1). This diagram only includes 'raw variables' from which BSI/AMR indicators can be computed. Having this homogenous data collection format at the source of the surveillance network will ensure that the same methodology is applied to all countries.

Figure S1. Data collection structure

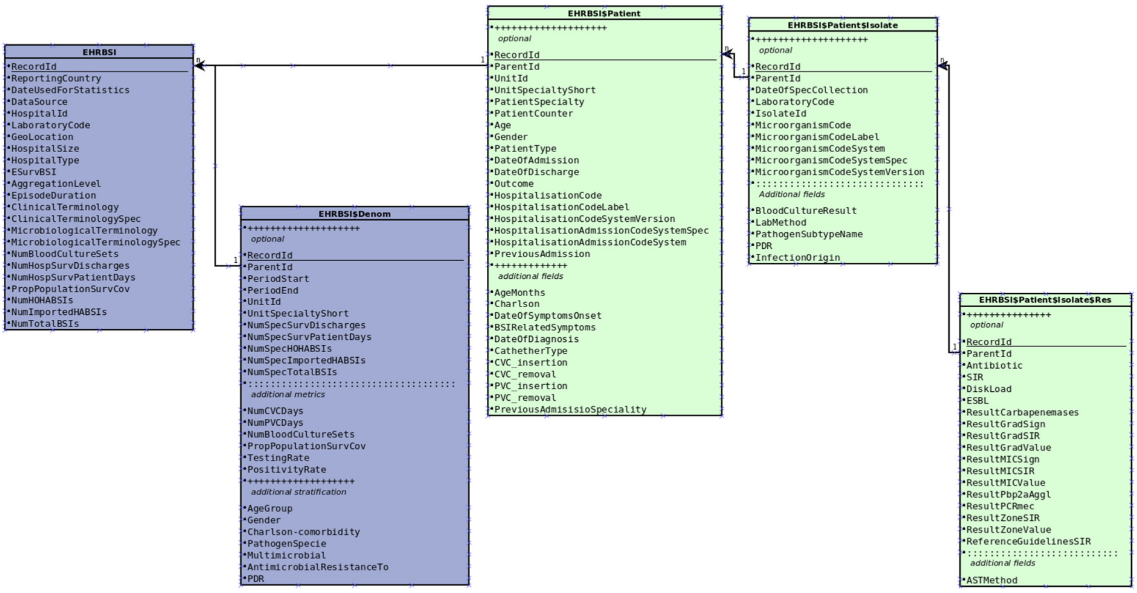

2. Data management and analysis

2.1. Type of analysis

In order to accomplish the four surveillance objectives (see section 1.1, aim and objectives), we consider different types of analysis:

- A retrospective cohort in which patients are followed-up and outcomes recorded over the study period to calculate an incidence rate based on a time-dependent denominator (person-time);
  OR
- A retrospective cohort study in which outcomes in patients are recorded and a cumulative incidence is calculated over the study period based on the population denominator at the start of the study period. This study design does not take person-time into account;
  OR
- A retrospective cohort where only the number of alerts of single pathogens defined as Emerging microorganisms (see section 1.4.3) or as pan-drug-resistant microorganisms are taken into account (eventually, incidence can be provided if the number of BSI episodes allows).

2.2. Data extraction

As described in section 1.3 (data collection period), although the aim for the future is to have a real-time single dataset, initially the countries can start working with different datasets and study periods per objective. The protocol envisages that to achieve the objectives of surveillance and alert of possible pan-drug resistance and emerging pathogens (objectives 2 and 3) these are best implemented as real-time, or nearly real-time as possible, mostly using laboratory datasets.

In contrast, achieving the objectives of measuring the incidence of HA-BSI and establishing electronic reporting of AMR according to the EARS-Net protocol (objectives 1 and 4) will benefit by the use of defined study periods and data extractions. Countries will run script(s) to compute the different indicators and this may require using different data sources (e.g. clinical, laboratory, hospitalisation etc) and more complex computations to define episodes and other variables. The data extraction for these objectives will require a download of data from X previous days to define the episodes (see section 1.4, key definitions, episodes definitions), which means that data will need to be extracted for a longer time period than the reporting period. For example, to report results for January to March 2023, data will need to be extracted for both periods before January (e.g., December 2022) and after (e.g. April 2023) the quarter in order to deduplicate episodes and allow data consolidation for key outcomes (e.g. death).
We recommend that sites perform an analysis of the time needed for data consolidation, for example, by comparing the number of BSI episodes in the same fixed period in different data extractions.

2.3. Denominator

Two types of denominators can be collected and reported to ECDC, although the provision of hospital rather than population denominators is recommended:

- Population denominators: related to the population of a region or the catchment population of a hospital, to calculate incidence in general population.
- Hospital denominators: to calculate incidence among all the patient admissions. It is collected at the level of ward/hospital/region/country or by patient characteristics (e.g. age groups or sex) and consists of the number of discharges/admissions or of patient-days. Patient-days are the number of days admitted at hospital, counting as a different day from 12 am. Transfer to another hospital is not considered a discharge.

Study sites/countries can choose to collect denominators for other relevant subgroups, such as age-groups, sex, geographical regions, clinical characteristics etc. for their own use.

2.4. Censoring events (only in case-based analysis)

All individuals (all admitted patients or the entire population) with an episode of BSI will be followed from the start of the observation period (see data collection period, section 1.3) to:

- Death of any cause (on the date of death).
- Discontinuation in the administrative database (i.e., emigration).
- End of observation period (depending on the country specific observation period, e.g., monthly or quarterly or others).

2.5. Data checking and validation

The following data checking and data validation should be undertaken before analysis:

- Identification of inconsistencies.
- Checking unusual values and outliers, including alert microorganisms and resistance patterns.
- Inclusion/exclusion criteria adherence.
- Missing data for essential variables that can lead to exclusion of the records from the analyses.
- Missing values, missing clinical details, missing laboratory results.
- Duplicate cases and multiple admissions.
- Consistency among dates (specimen collection, admission, discharge, death).
